# Challenges for the balanced attribution of livestock’s environmental impacts: the art of conveying simple messages around complex realities

**DOI:** 10.1093/af/vfac096

**Published:** 2023-04-15

**Authors:** Pablo Manzano, Jason Rowntree, Logan Thompson, Agustín del Prado, Peer Ederer, Wilhelm Windisch, Michael R F Lee

**Affiliations:** Basque Centre for Climate Change (BC3), E-48940 Leioa, Spain; Ikerbasque — Basque Foundation of Science, E-48007 Bilbao, Spain; Department of Animal Science, Michigan State University, East Lansing, MI, USA; Department of Animal Sciences and Industry, Kansas State University, Manhattan, KS, USA; Basque Centre for Climate Change (BC3), E-48940 Leioa, Spain; Ikerbasque — Basque Foundation of Science, E-48007 Bilbao, Spain; Global Food and Agriculture Network, Rapperswil, Switzerland; Technical University of Munich, Liesel Beckmann Straße 2, D-85354 Freising, Germany; School of Sustainable Food and Farming, Harper Adams University, Edgmond, Newport, Shropshire, TF10 8NB, UK

**Keywords:** biodiversity, circular agriculture, global warming potential, life cycle assessment, methane, water

**Implications**

- Meat production is often listed among the largest contributors to climate change, and is usually associated with biodiversity damage, feed-food competition, and water scarcity. This assumption is largely based on the biogenic methane (CH_4_) emissions of the global herd of ruminants and its occupation of land. Environmental assessments of the livestock sector are all too frequently stated in simplistic terms, making use of a myopic selection of metrics, and overlooking underlying heterogeneity and complexities.
- One example of such oversimplification is the comparison of the warming effect of different greenhouse gases (CO_2_, CH_4_, and N_2_O), which are associated with a series of challenges due to their own heterogeneous atmospheric ‘behavior’. Whilst useful for certain research questions, standardizations such as the commonly used GWP_100_ hide many complex issues. These issues include considering different emission profiles of production systems (e.g., low-methane porcine vs. high-methane ruminant), the need to factor in CO_2_ and CH_4_ sinks, the different atmospheric lifetimes of each gas and subsequent atmospheric warming potential, and compensatory background emissions in alternative rewilding scenarios.
- Whilst poorly managed land negatively affects biodiversity, well-managed land strategies, including those pertaining to livestock production, can lead to favorable outcomes (e.g., biodiverse swards that encourage pollination and beneficial microfauna). Similarly, the assessment of water wastage and land use requires contextualized approaches. This highlights the importance of addressing agricultural heterogeneity in systems analysis, including Life Cycle Assessment (LCA).
- To further reflect the food-environment nexus, nutritional LCA (nLCA) incorporates considerations of food. optimizing e.g. nutritional sustenance and reducing, in theory, the amount of food we consume through meal-level assessment - rather than focusing on a single product.• Being more recent than the wider LCA ‘umbrella’ (e.g., Life Cycle Cost Analyses), one current drawback of nLCA is that it can be easily manipulated to favour one product over another, whether plant- or animal sourced, by singling out specific nutrients (e.g., fiber or vitamin C vs. vitamin B12 or digestible amino acid balanced protein).
- When considering the value of livestock products against their environmental impact, a holistic assessment is needed using balanced metrics and avoiding tunnel vision. Besides factoring in nutrition and co-product benefits, other natural capitals, and societal assets that result from well-managed farm enterprises need to be acknowledged, even if no empirical metric can currently fully account for their true value. Examples include: biodiversity, soil health, land stewardship, and rural community support; especially in a time of extreme variability due to climate, social unrest, and economic crises.

## Introduction

A major challenge for the scientific community has been the development of balanced metrics to evaluate the environmental, social, and economic impacts of livestock production systems, which enable feasible policy action scenarios that balance the protection of natural capital with food security. For instance, the difficulty of robustly assessing the relative impact of various livestock-associated greenhouse gases (GHGs) on the climate provides a clear example, especially given the vast differences in behavior and variable lifetimes in the atmosphere of the individual GHGs, and how this relates to short- and long-lived climate agents. For industries such as ruminant agriculture, where the primary emissions are non-CO_2_ (i.e., methane, CH_4_, and nitrous oxide, N_2_O), the way these metrics equate to CO_2_-equivalents (CO_2_-eq) has overly simplified their impacts on global warming, compared to industries that primarily emit fossil fuel-sourced CO_2_. Other environmental impacts, such as the degree to which livestock production uses water, which subsequently is not available for other human uses, or the effects that land use has on cropland scarcity or biodiversity, have suffered from similar issues of misrepresentation through oversimplification. In tandem with the aforementioned complexities of sustainability assessments, identifying metrics that represent a food’s nutritional value vs. just using units of mass, protein or energy have been developed to better elucidate the true nutrition-environment  nexus.

The goal of this paper, therefore, is to outline current issues related to the quantification of livestock’s impacts on the environment, briefly describing alternative metrics for more transparent, and holistic impact accounting. Furthermore, it is argued that accounting for single environmental impacts ignores the broader value of livestock, and other agricultural commodities for that matter, as part of a circular food system that contributes to social resilience beyond one major anthropogenically driven challenge, such as climate change. Keeping these other aspects out of the scope of consideration will likely invite unexpected and highly negative consequences that would then backfire on any progress otherwise made.

## Complexities Related to Accounting for Biogenic Methane’s Impact on Climate

With respect to climate change, carbon footprints’ impact assessments usually adopt GWP_100_ characterization factors (i.e., global warming potential over a 100-year time horizon), thus standardizing the atmospheric effects of all GHGs to CO_2_-eq. It is typically claimed under GWP_100_ that CH_4_ is a GHG 28 times more potent than CO_2_. The origin of this number is the Intergovernmental Panel on Climate Change’s (IPCC) Assessment Report (AR) 5 published in 2013 (IPCC, 2013). In the IPCC AR 6 (IPCC, 2021), which replaced AR 5, the number was refined to 27.2 for biogenic CH_4_ sources of non-fossil origin. IPCC (2021) now explicitly recommends sensitivity analyses of timeframes considered to better represent the complexities of various GHG’s atmospheric behavior. For instance, if calculated under a 20-year timeframe (GWP_20_), a CH_4_ (non-fossil) is considered to have a GWP 80.8 times more potent than CO_2_, whilst over 500 years (GWP_500_), it is 7.3 times more potent than CO_2_. These IPCC precise published values (to 1 decimal place) suggest an accuracy of understanding of atmospheric dynamics, which in reality is not available. However, the more recent standardization factors and impact assessment advice published under AR 6 (IPCC, 2021) do provide recommendations for the calculation of CO_2_ uptake, taking a step forward in acknowledging carbon cycling response (formally referred to as carbon feedback), both positive and negative depending on the system under investigation. If reported accurately and transparently, this is one way of mitigating subjective decision-making related to sustainability assessments (as will be elucidated in the next section).

## Greenhouse Gas Carbon Dioxide Equivalents, Inherent Weaknesses, and Novel Solutions

When converting the greenhouse effect of various GHGs to CO_2_-eq, complexities emerge due to differences in their decomposition or removal (sink) characteristics from the atmosphere. In brief, CH_4_ decomposes mostly to CO_2_ and H_2_O in the atmosphere within a few years (Lelieveld et al., 2016). This decomposition happens primarily through reaction with hydroxyl (OH^-^) radicals (Li et al., 2008), often nicknamed the detergent of the atmosphere because they also react with a number of other atmospheric gases and thus “clean” the atmosphere of otherwise damaging buildups of various chemical elements. This creates a highly complex chemical reaction scheme, which is as yet insufficiently understood by the atmospheric sciences. In contrast to CH_4_, CO_2_ is highly inert and reacts minimally in the atmosphere. It thus requires terrestrial and aquatic sinks to be removed, which function predominately through photosynthesis, or dissolution in oceans (causing increased acidification). Since both the photosynthetic and oceanic capture cycles are in long-term equilibrium, additional injections of CO_2_ from fossil fuel sources outside of these cycles gradually accumulate and deposit in the atmosphere without the prospect of dissolution within human-relevant timescales. N_2_O, a potent GHG emitted from agricultural systems, not discussed in detail here, would behave as a long-lived gas in the context of a GWP_100_ metric. However, for the purpose of this discussion, its behavior would be intermediate between CO_2_ and CH_4_. The different atmospheric dynamics of these GHG’s, CO_2_, N_2_O, and CH_4_, need to be reflected in climate change considerations, a practice rarely conducted by sustainability analysts (Lynch, 2019), particularly when policymaking in relation to agricultural climate action.

As with all models, climate change models are prone to uncertainties through the inherent simplification of complex biochemical processes. Despite GHG measurements and subsequent calculations becoming more sophisticated as technology improves, such models still suffer from a lack of granular primary data on the one side, and a tendency of complex systems (e.g., the carbon cycle) to reach tipping points where system dynamics undergo rapid changes (e.g., change of albedo following ice cap melting) on the other. With these challenges in mind, predicting the true effect of complex nutrient cycles (carbon in this case) on the atmosphere becomes a daunting task. However, until more primary data becomes available to improve existing characterization factors related to metrics such as GWP_100_, there are some measures scientists and sustainability assessors can take to increase transparency related to the effect of their subjective decisions (e.g., choosing one GHG impact assessment over another). For example, when reporting GWP_100_ values, it is prudent to also report impacts under GWP_20_ and GWP_500_. In addition, it is advisable, particularly when using the carbon footprint/LCA framework, to test the uncertainty of emission factors that drive the total amount of GHGs produced in a given system, whether carbon- or nitrogen-based, in the first place (e.g., CH_4_ conversion factors, known as Y_m_ under IPCC guidelines, and emission factors which determine how much nitrogen is lost to the environment as ammonia (NH_3_) or N_2_O, for example). Furthermore, acknowledging both the likely sinks of CH_4_ and hence its more rapid removal (e.g., bacterial methanotrophs in soil and atmospheric OH^-^ radicals) compared with long-lived GHGs (e.g., CO_2_ and N_2_O), and uncertainties associated with relevant sink processes provide greater insight into physical and biochemical atmospheric processes.

The fact that the vast majority of atmospheric CH_4_ removal comes from the OH^−^ sink, interlinks the climate impact of CH_4_ with prevalence and regional distribution intensities of other gases in the atmosphere such as carbon monoxide (CO) or volatile organic compounds (VOC) which are also subject to the same OH^-^ sink removal. Either overall or regional changes in the concentrations of any of these gases can have reinforcing or dampening effects on the atmospheric chemical reaction system, depending on a variety of circumstances. Some models suggest that small changes in the resultant OH^-^ availability can lead to large changes in the residence time and radiative forcing of CH_4_ and therefore, on the way, CH_4_ emissions will affect CH_4_ concentrations in the atmosphere (e.g., stagnation of CH_4_ concentrations in the period 1999–2006; McNorton et al., 2016). Other models describe that the OH^-^ system has substantial buffering capacity, which suggests that the impact on CH_4_ buildup and removal could be regionally driven, rather than universally driven (Lelieveld et al., 2016). Most climate atmospheric models assume that OH^-^ availabilities are time invariant (Turner et al., 2017), but in real conditions, the presence of OH^-^ is likely to vary depending on the concentration of gases that typically react with it (e.g., CO or VOC) and, which have been shown to vary in time (e.g., a steady fall of CO emissions) or/and are sensitive to climate change events (e.g., increased temperatures or fires; Boy et al., 2022). It must therefore be acknowledged that the processes behind OH− sink variability are still poorly represented and under scientific debate (Turner et al., 2017) with an urgent need to be addressed, so that understanding of CH_4_ budgets can be improved.

With a history of CO_2_-eq criticisms (Pierrehumbert, 2014), there have been numerous attempts at developing alternative metrics to GWP, some, such as Global Temperature Change Potential, GTP_X_, which bestows a much lower characterization factor for CH_4_ (6 times more potent than of CO_2_) are now included in IPCC reports (IPCC, 2021). A further metric, GWP*, has recently been developed that converts CH_4_ emissions into ‘CO_2_-warming equivalents’ (CO_2_-we; Allen et al., 2018, Cain et al., 2019a). With a strong correspondence to mechanistic climate modeling, this metric is argued to more aptly represent how CH_4_ emissions translate into temperature outcomes at various points in time by treating this gas as a flowing gas rather than a stock gas like CO_2_. Different studies that have developed, improved, or used the GWP* metric at different scales and with different frameworks (cumulative emissions *vs.* pulse emission) are shown in Table 1. One particularly useful application of GWP* when analyzing the impact of future global scenarios of GHG emissions on additional global temperature, is by calculating warming equivalent emissions and relating these emissions (e.g., expressed as a cumulative way) with the additional warming caused from a reference year. This is analogous to the way net-zero has been estimated for CO_2_ and N_2_O emissions, considering that each long-lived GHG (CO_2_/N_2_O) molecule emitted can be thought of as raising temperatures in a straightforward, additive manner. The warming contribution of CO_2_/N_2_O can be determined by summing all their past emissions to date, which is not the case with short-lived or flow gas GHGs such as CH_4_. In this sense and when using cumulative GWP*, Costa et al. (2022) and Liu et al. (2021), reported that reducing global livestock CH_4_ emissions by 7% from 2020 to 2040 (at 0.35% annual reduction in emissions) would stop further agricultural CH_4_-related increases in global temperatures—analogous to the impact of net-zero CO_2_ emissions (as explained by Allen et al., 2022). Furthermore, reducing emissions by 5% annually over this same time horizon would neutralize warming that had occurred since 1980. However, if CH_4_ emissions were to rise by 1.5% annually, the GWP* method resulted in a 40% greater climate impact than if CH_4_ emissions had been converted to CO_2_-eq using GWP_100_. This, therefore, highlights that the metric is not “livestock friendly” under all conditions, as often perceived, as increases in emissions would make the livestock industry even greater contributors to the global GHG budget than the status quo. The GWP* metric has recently been also applied, beyond cumulative emissions, to pulse ones (Table 1), e.g., to calculate C footprints in livestock products in New Zealand (Mazzetto et al., 2023). The value of C footprinting calculated using GWP* expresses the relative warming added by CH_4_ emissions compared with a reference year 20 years before.

**Table 1.** Main issues covered by the different studies developing, improving, or using the GWP* metrics

Main questions covered	Applied scale	Cumulative/pulse emissions	Specific mitigations?	Studies
How GWP* methodology was developed/improved and basic use for reporting global contributions to warming	Global	Cumulative	No	Allen et al. (2018, 2021); Cain et al (2019a); Lynch et al. (2020, 2021); Smith et al. (2021)
How much warming does an individual’s lifetime diet cause	Country, diet	Cumulative	yes	Barnsley et al. (2021)
How much warming in relation toNDC and Paris Agreement	Global, Food systems	Cumulative	yes	Cain et al (2019a, 2019b); Clark et al. (2020); Costa et al. (2022)
How much agricultural CH4 emissions scenarios increase global temperatures and potential future reduction through measures	Global, Continental, Country	Cumulative	yes	Costa et al. (2021); del Prado et al. (2021); Liu et al. (2021); Hörtenhuber et al. (2022)
What the effect on the efficacy of CH4 mitigation options is of using a particular climate metric affecting methane’s warming potential	Global	pulse	yes	Pérez-Domínguez et al. (2021)
What the C footprint of livestock products is (relative warming caused as a consequence of changes in CH4 intensities in 2 dates varying 20 years)	Country	pulse	yes	Ridoutt (2021); Ridoutt et al. (2021, 2022); Mazzetto et al. (2023)

**Figure 1.**
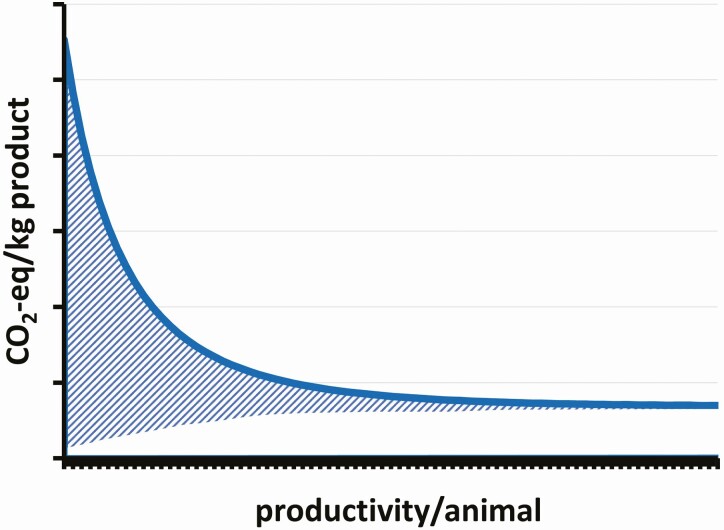
Theoretical curve displaying the range of GHG emissions potentially attributable to a natural GHG ecosystem baseline (striped surface), applied to the conceptualization in Gerber et al., (2011)’s Figure 5.

As a final consideration, natural baselines are key to the climate change discussion. This has not only implications for product comparison assessments, but also with respect to what is considered “natural” within ecosystems. Usually, farmed livestock emissions are considered as anthropogenic. Yet, this assumption ignores how ruminant management integrates itself in grazing ecosystems, which predate the existence of livestock by many millions of years. While grazing ecosystems occupy vast expanses of Earth’s terrestrial ecosystems, they are currently used for crops or animal husbandry in most of their extension. When such lands are abandoned, as has happened after, e.g., the Chernobyl disaster, wild herbivores re-occupy the grazing niches, emitting CH_4_ that is in turn considered a natural ecosystem flow. However, this exemplifies that the abandonment of grazing livestock, and the subsequent ecosystem changes that follow (e.g., loss of habitat for ground-nesting birds; Pearce-Higgins and Yalden, 2003), is not as effective as a global warming mitigation strategy as has been claimed (Manzano & White, 2019), as the balance from domestic herbivores disappearing from the landscape is not zero. This will be particularly significant in some developing countries with well-conserved herbivore guilds that achieve high biomass concentrations when undisturbed, such as East Africa or South Asia (Fløjgaard et al., 2022). Alternative scenarios without large herbivore guilds imply higher termite abundances or more frequent and intense wildfires, both cases also having the capacity to generate large amounts of CH_4_ and CO_2_, respectively. A balanced accounting of livestock’s climatic impacts should discount such natural background emissions (Figure 1) from those currently attributed to animal husbandry (see Pardo et al., 2023 for a first analytical approach) and account for the elevated risk of wildfires if they were withdrawn (which is increasing in the face of climate change). In addition, tropical grass-based livestock systems with extremely low inputs also seem to need refinements in the CH_4_ emission factors they are assigned, which seem to be significantly lower than expected from previous assumptions (Pelster et al., 2016; Assouma et al., 2017). Similarly, N_2_O emissions are lower from extensively-managed upland grasslands than from intensively managed grasslands (Marsden et al., 2018) and when forage legumes are present than when they are absent (McAuliffe et al., 2020a).

## Factoring in Nutrition and  Co-product Benefits

Current metrics that evaluate agri-food production under a mass or volume-based ‘functional unit’ (LCA terminology for a denominator by which impact categories (e.g., carbon footprint, CO_2_-eq) is the numerator, to allow comparability) do not necessarily reflect the nutritional value (content and availability) nor socio-geographic context of food. Whilst mass and volume may be appropriate in certain circumstances (e.g., similar farming practices for the same commodity; Lee et al., 2021a), when comparing food items with varying nutritional properties, the ‘function’ of that food should be accounted for: namely, to provide human sustenance and support health as usually defined in recommended daily intakes of critical nutrients (McAuliffe et al., 2018). To achieve such a nuance, nutritional LCA (nLCA) has been developed over the last decade (e.g., Saarinen et al., 2017; Sonesson et al., 2017; Sonesson, 2019). nLCA is a reasonably novel approach falling under the LCA framework which integrates nutritional and, in some cases, health metrics into the modeling process (McAuliffe et al., 2020b). It has received considerable attention, as demonstrated by an FAO report recently published by an international consortium of LCA experts, in addition to epidemiologists, nutritional scientists, and health scientists (McLaren et al., 2021). The FAO report not only highlighted the benefits of livestock systems, which provide a highly bioavailable source of key nutrients (i.e., lean, unprocessed animal-based products) but also highlighted limitations both of certain processed foods (including animal-sourced foods) and of the method itself.

One of the major limitations of nLCA is the lack of data pertaining to food bioavailability and digestibility, which can differ drastically between certain plant-based products (i.e., some plants have ‘anti-nutritional factors’ which prevent the uptake of various nutrients, whereas animal-based products have virtually 100% bioavailability and digestibility). Whilst numerous authors have addressed this issue (McLaren et al., 2021), the reality is that in vivo proxies need to be used (i.e., rats and pigs, which have similar digestive tracts to humans) to estimate the bioavailability of individual nutrient-uptake in humans. Furthermore, and perhaps more complex than purely bioavailability, one of the most commonly used ‘nutritional functional units’ is a protein (e.g., reporting environmental footprints on the basis of 100 g protein), as this nutrient is not a single homogenous compound equal across all food items. Protein is made up of 20 amino acids (21 including selenocysteine), including indispensable amino acids (IAAs) which can only be sourced from food and are required for protein accretion. There are scoring mechanisms in place such as the Digestible Indispensable Amino Acid Score (DIAAS; Marinangeli and House, 2017) which provides a value of total digestibility of the IAA, and these scores can be over 100% for some animal-based products (e.g., milk and certain lean meats), whilst some plant-based products can be around 45% (e.g., cereals such as wheat). Without accounting for the digestibility and bioavailability of nutrients, including IAA, comparisons using protein in LCA are largely inconclusive (Moughan, 2021), yet rarely achieve such recognition due to a current lack of interdisciplinary collaboration between the fields of environmental and nutritional/health sciences.

On a more positive note, nLCA has been used to innovate entirely new ways of looking at comparisons between food items. For example, some authors have used nutritional density scores (NDS) which provide scalar values for the nutritional benefits of individual foods. The most commonly used NDS is known as Nutrient Rich Food (NRF) 9.3 (Fulgoni et al., 2009). NRF calculates the ratio of nutritional composition of foods versus the recommended daily intake (for encouraging nutrients) and allowance (for limited nutrients). However, the included ‘encouraged’ (protein, fiber, vitamins A, C, E, calcium, iron, magnesium, and potassium) and limited nutrients (saturated fat, added sugar, and sodium), still do not provide a complete set of essential nutrients e.g. vitamin-K, B-vitamins and selenium. This has led authors to develop their own NDS by including up to 22 nutrients in some cases to gain a wider insight into the overall benefit, or disbenefit, of a food item (McAuliffe et al., 2020b; Lee et al., 2021a, 2021b).

Alongside NDS scores and DIAAS-corrected protein functional units, other metrics are being developed to link all processes along a supply chain–thereby estimating burdens of a given impact category such as carbon, eutrophication, acidification potentials, and land occupation to connect and compare environmental impacts from a food product with the combined health impact based on both the losses to nature (e.g., particulate matter which affects the respiratory system) and the health impacts from consuming the same food. This is an entirely novel way of looking at nLCA and is in its infancy. However, this novel method is gaining traction and despite the complexity involved, many groups are adopting, improving, and interpreting this new way of looking at the food-health-environment nexus (McLaren et al., 2021). One such complexity which requires attention is the trade-off between environmental impacts and human nutrition, which is often subjectively weighted. This has resulted in curious claims that sweets (e.g., certain candies) score higher than lean meat and even eggs, the reference product for protein-source foods (Stylianou et al., 2021). This paper has undergone considerable criticism and is currently being discussed within the realms of LCA of nutritional science, particularly due to its reliance on the Global Burden of Disease, a database with spurious assumptions and omissions about food composition and subsequent impacts on health (Stanton et al., 2022). Moreover, the dangers of developing carbon labeling are obvious, as sugars and syrups with low CO_2_-eq footprint would stimulate acquisition by consumers in spite of their low nutritional value. More subtle dangers are hidden with animal-sourced foods with high nutritional value and high CO_2_-eq footprint but that ignores elements discussed here, such as the non-anthropogenic nature of part of such GHG emissions or the positive land use effects of grazing livestock. Simple carbon labeling can therefore potentially stimulate consumption patterns that are neither good for the nutritional status of consumers, nor for the environment.

In addition to nutritional value, the way LCAs of animal-derived foods are often interpreted as neglect to equitably allocate portions of the emissions profile with the non-edible co-products and services associated with their production (e.g., hides, wool, fats, organs, milk, bone, serum, manure, draught power, pet food, pharma, etc.). This is in spite of examples of LCA trying to find the best ways to allocate non-tangible benefits, e.g. draught power or cultural status (Ripoll-Bosch et al., 2013, Weiler et al., 2014). Moreover, livestock is known to provide other important benefits, such as social status, access to capital, opportunities to fund education and health services, or elements necessary to female emancipation, which interact with each other in complex ways. The need to develop specific indicators for this (Manzano et al., 2021) reveals also a necessary integration with carbon footprint analyses that, albeit complex, should be in the viewpoint of research agendas. Appropriate allocation is required to account for all the functions of co-products in their various uses and markets, which is a complex task, especially given the differences that are encountered when contrasting economic and mass allocation models (Le Féon et al., 2020).

## Balancing Impacts Related to Land Use, Water Wastage, and Biodiversity

When assessing the impact of ruminant livestock, it is important to acknowledge that this refers—for a very substantial part—to the valorization of nonproductive land, i.e., land that is not suitable for arable cultivation. Crops are considered to utilize 12% of the total land area, while livestock is considered to valorize a further 37% (Arneth et al., 2019), although this does not consider the low-intensity use by livestock of forest (Manzano, 2015) and of other lands considered to be subjected to minimal human use. This is materialized in the production of high-quality foods from byproducts (from the food industry) and land not suitable for growing crops, (Wilkinson and Lee, 2018; Lee et al., 2021b) as well as in the supporting of rural communities, protection of natural capital, and maintenance of biodiversity. One way of categorizing this up-cycling of industrial byproducts and nonproductive land to produce highly nutritious food is through the Net Protein Contribution (NPC) of a food production system. For instance, because ruminants have considerably less feed-food competition than monogastric livestock, they upcycle 3-4-fold more NPC to the human diet than pork or poultry (Place & Myrdal Miller, 2020). Furthermore, ruminant livestock also fulfills a vital role in subsistence farming in the developing world, through a provision of financial and climate resilience (Eisler et al., 2014).

Livestock, particularly when hosted on grazing landscapes in temperate climatic conditions, are often questionably attributed with large water footprints according to accounting methodologies that have become popular. One example is a footprinting approach which includes all sources of water regardless of their depletion of natural capital (these sources are often referred to as green, blue, and grey water). Widespread confusion around water accounting has driven the FAO to facilitate a consensus process among the water footprint researcher community to interpret such metrics (Boulay et al., 2021). Whilst, of course, there are exceptions depending on geography and local water resources, in many such calculations, the quantity of water attributed to livestock systems, particularly grassland-based, is dominated by the green water fraction (equivalent to the amount of rainwater that falls on the lands being grazed). It is, however, contentious to count green water in water footprint evaluations. It is a metric rather similar to land use, with little practical use to estimate water scarcity or the degree of competition for water between livestock and humans. Little rainwater falling on grazing lands will be removed by livestock from the system. Rather, the vast majority will infiltrate the ground to recharge underground water stocks or flow to feed streams—both being sources for the “blue” water that food-producing or industrial activities, and water supplies, compete with. The paradox of such a water accountancy method is that, in rainy mountainous areas with high slopes and strong water flow, shallow soils, and negligible potential for crop agriculture, local livestock will be attributed with a very large water footprint, yet with negligible impacts of water scarcity and availability for other uses. This is not to say that, in certain circumstances, livestock cannot be intensive consumers of ‘blue’ water—hyper-arid areas, for example, where livestock production needs to make use of channeled water, or irrigated crop production for feed will undoubtedly have created groundwater exhaustion. Consensus among researchers points to water scarcity measurements being much more effective in describing the efficiency of the use of water resources and recommends to never neglecting this aspect. As described above in the context of GHGs, however, when there is not a detailed understanding of a system or a clear objective choice for a particular methodology (e.g., if green, blue, or grey water consumption is uncertain, yet total water usage is known), sensitivity analyses should be conducted to report a range between best case scenario and worst-case scenario. It would also be prudent to report different impact assessments (e.g., water scarcity versus water footprinting in the case of water consumption). Without robust objectivity underlying sustainability assessments, which is rare given the complexities described above, scenario analyses are arguably the most scientifically sound approaches to account for modeling weaknesses. Media should therefore correct their messages to reflect real societal issues around competing water uses, and not methodological artifacts void of practical significance.

As described elsewhere in this Special Issue (Thompson et al., 2023), uses of land for ruminant production are not necessarily negative for biodiversity or ecosystem functionality. They can have large positive outcomes (Teillard et al., 2016), including the increase of carbon stocks in soils, and especially if they mimic the behavior of the wild herbivores that have shaped most of the planet’s ecosystems in the last 12–15 million years (Manzano et al., 2023). Soil carbon, however, is not stored permanently. Turn-over of soil organic matter occurs continuously over a range of timescales and is sensitive to management and climate factors, resulting in some soils being a net source or a net sink of organic carbon (Smith, 2004, 2005). The challenge is to identify soils that have been depleted of carbon by farming practices (for example, intensively cultivated arable mineral and organic soils) or natural events that will be responsive to restoration by management that fosters soil carbon repletion, such as return of animal manures or grassland rotations (Smith, 2014). There are undoubtedly some circumstances in which carbon sequestration can be used to increase soil carbon storage, especially in depleted arable soils that have a high potential to store more carbon. Permanent grassland soils, however, will approach an equilibrium state as they age in which the quantity of carbon gained is equal to carbon losses (Smith, 2014; Sanderson et al., 2020). However, the period to reach this equilibrium will depend on many soil and management factors. As many grassland soils are relatively rich in organic carbon when compared to those elsewhere, there may be challenges but also opportunities to manage these soils associated with maintaining or increasing existing soil organic carbon stocks (Smith, 2004). For grasslands that may have reached equilibrium, grazing management will play a vital role in maintaining these carbon stocks as the main terrestrial carbon store (Soussana and LeMaire, 2014).

Adding to the benefit of soil as a carbon capture approach, increasing soil carbon also improves overall soil health (biological functioning) and water-holding capacity through improved physical microscale structure. Limiting organic carbon inputs and tillage degrade this structure and the hydraulic conductivity and water holding capacity are reduced consequently (Neal et al., 2020). In high-carbon, well-structured, and more oxygenated soils such as grasslands, microbes assimilate nutrients into biomass more effectively and nutrients are therefore retained in soil rather than lost. The increased water-holding capacity of high-carbon soils, typical of grazed grasslands also has practical implications for reducing flood risks, a vital service of our ruminant grassland systems (Neal et al., 2020).

Taken together, a balanced accounting of land use and biodiversity needs to account for scarcity of lands suitable for crop production, much in parallel with water use accounting, and needs to properly discriminate between positive and negative environmental outcomes of different livestock practices and the vital role they can play in maintaining and restoring soil health.

## Conclusion

The complexity of environmental impact accounting typically leads to over-simplistic use of an impact metric, e.g., CO_2-eq_/kg product or unit of protein/energy, which does not represent the true impact and value of livestock products. It is flawed on both sides of the equation: CO_2_-eq does not adequately reflect the different nature of CH_4_, the main GHG emitted from ruminant livestock systems, compared to CO_2_ and N_2_O in the atmosphere. On the other hand, kg product does not adequately consider the value of livestock: for example, nutritionally, they are generators of valuable co-products, whilst also being re-cyclers of byproducts, up-cyclers of nonproductive land, potential soil and biodiversity enhancers, and also offer social resilience platforms. However, there are alternatives that, even if not perfect, better reflect the value proposition of animal-based products. They can, for instance, consider the natural turnover of CH_4_ compared to CO_2_/N_2_O (cf. GWP*), or the nutritional value of the food produced in terms of human health (cf. nLCA).

However, even if not satisfactory from a pragmatic perspective, the reality is that a single metric will never do justice to the complexity of the various livestock production system impacts. While some degree of simplification is inevitable, a multifactorial assessment approach will usually be necessary. Ideally, metrics should aim at accounting for the wider value of livestock in our food system, providing opportunities for biodiversity (through appropriate stewardship), restoring soil health, reducing the risk of wildfires, and supporting rural communities at a time of climate uncertainty. Yes, the challenges of reducing GHGs are relevant to all sectors including livestock, but balanced burden attribution needs to be applied to ensure the outcome is not perverse when considering the needs for feeding a growing population and the value livestock so clearly provides. Radical actions based on unbalanced metrics can also greatly impact livestock systems that have a large valuable contribution to rural livelihoods, especially in the Global South. Livestock-depending subsistence and smallholder farmers, numbering ca. 1 billion (Robinson et al., 2014), would be negatively impacted by any simplistic actions that significantly reduce the livestock systems currently assumed to be more damaging for the climate, triggering a cascade of social effects with unpredictable consequences.

## About the Author(s)

**Pablo Manzano** works as an Ikerbasque Fellow at the Basque Centre for Climate Change in Bilbao, Spain. A rangeland ecologist, he graduated from the Autonomous University of Madrid and has gathered research experience at the FSU Jena, Germany, and University of Helsinki, Finland, plus shorter stays at Landcare Research, Christchurch, New Zealand, and Texas State University, San Marcos, USA. In parallel, he has developed a career in international development cooperation, with experience at several NGOs as well as international organizations such as IUCN, FAO, or the World Bank. His experience has allowed him to perform transdisciplinary research on livestock-based socio-ecosystems.

**Jason Rowntree** is a professor of Animal Science at Michigan State University where he holds the Charles Stewart Mott Distinguished Professorship for Sustainable Agriculture and is the Co-Director of the newly formed MSU Center for Regenerative Agriculture. Rowntree’s research focus is identifying the metrics and management that reflect ecological improvement in grazing land systems. Rowntree has worked to co-develop, with the Savory Institute, an Ecological Outcome Verification that is now being used on over 2 M acres of grasslands on every continent except Antarctica.

**Agustin del Prado** is an Ikerbasque Associate at the Basque Centre for Climate Change (BC3). An Ecosystems Biologist (1997) with a PhD in Biological Sciences (2007) from the University of the Basque Country (Spain), after post-doc and senior researcher positions at IGER (UK) (now Rothamsted Rs) (2002-2008), he became Research Professor and group leader at the BC3. He is an expert in the development of mathematical simulation models to assess and mitigate the effect of ruminant farming on climate change, resulting in work as an IPCC Lead Author (2019 Refinement to the 2006 Guidelines for National GHG Inventories). After being its first president (2012-2017), he is a promoter of the Spanish Science Network REMEDIA on climate change mitigation.

**Logan Thompson** is an Assistant Professor at Kansas State University in Manhattan, Kansas. He completed his bachelor’s degree at Texas Tech University in 2015. Following this, he earned a Masters in beef sustainability at Oklahoma State University in 2017 and a PhD in ruminant nutrition at Michigan State University in 2021. Prior to joining Kansas State University in August of 2022, he served as a post-doctoral fellow at Colorado State University. Logan’s predominant research interest is in measuring greenhouse gas emissions in grazing systems and how grazing management impacts ecosystem function.

**Peer Ederer** is founder and director of GOALSciences, the Global Observatory of Accurate Livestock Sciences, which has the mission to research and communicate scientific evidence about the role of animals in the global food system. He has an MBA degree from Harvard University, a PhD in financial economics, and holds an adjunct professorship for innovation studies. He has extensive experience in strategy advisory to private companies and public bodies in the global food system and is a frequent presenter on related topics. He has been engaged in scientific research in cooperation with globally leading universities from around the world and is a member of the Scientific Council of the World Farmers Organization.

**Wilhelm Windisch** is full professor of Animal Nutrition at the Technical University of Munich (TUM), Germany, since 2010. As an agricultural scientist, he finished his doctorate in 1988 and his habilitation in 1995. Prior to his appointment at TUM, he was a full professor of Animal Nutrition at the University of Natural Resources and Life Sciences Vienna (BOKU), Austria (2002-2010). He studies nutritional properties of feed materials and principles of biomass transformation in the metabolism of agricultural livestock with a particular interest in minerals, functional dietary compounds including antinutritive substances, and the impact of livestock feeding on food quality, food safety, and the environment.

**Michael Lee FRSA** is a Professor in sustainable livestock systems, defining their role in securing global food security at the same time as protecting environmental health (Livestock’s role in human and planetary health). He graduated with first-class honors in Animal Science from University of Wales, Aberystwyth in 1997 and gained a PhD in ruminant nutrition (protein and energy metabolism) from the University of Aberdeen in 2001. He is currently Deputy Vice Chancellor of Harper Adams University, England’s Premier specialist agriculture, and land use University. Professor Lee is President of the European Federation of Animal Science—Livestock Farming Commission and co-chair of the UK Universities Network on Climate Change—Net Zero Group. He was President of the British Society of Animal Science and was awarded the Sir John Hammond Prize in 2015 for service to Animal Science.
